# Altered dynamic functional connectivity of motor cerebellum with sensorimotor network and default mode network in juvenile myoclonic epilepsy

**DOI:** 10.3389/fneur.2024.1373125

**Published:** 2024-06-06

**Authors:** Menghan Yang, Yingying Zhang, Tianyu Zhang, Huanyu Zhou, Jiechuan Ren, Dong Zhou, Tianhua Yang

**Affiliations:** ^1^Department of Neurology, West China Hospital, Sichuan University, Chengdu, Sichuan, China; ^2^Department of Neurology, Beijing Tiantan Hospital, Capital Medical University, Beijing, China

**Keywords:** juvenile myoclonic epilepsy (JME), motor cerebellum, dynamic functional connectivity (dFC), EEG-fMRI, default mode network (DMN), sensorimotor network (SMN)

## Abstract

**Objective:**

To investigate whether changes occur in the dynamic functional connectivity (dFC) of motor cerebellum with cerebral cortex in juvenile myoclonic epilepsy (JME).

**Methods:**

We adopted resting-state electroencephalography—functional magnetic resonance imaging (EEG-fMRI) and a sliding-window approach to explore the dFC of motor cerebellum with cortex in 36 JME patients compared with 30 and age-matched health controls (HCs). The motor cerebellum was divided into five lobules (I–V, VI, VIIb, VIIIa, and VIIIb). Additionally, correlation analyses were conducted between the variability of dFC and clinical variables in the Juvenile Myoclonic Epilepsy (JME) group, such as disease duration, age at disease onset, and frequency score of myoclonic seizures.

**Results:**

Compared to HCs, the JME group presented increased dFC between the motor cerebellum with SMN and DMN. Specifically, connectivity between lobule VIIb and left precentral gyrus and right inferior parietal lobule (IPL); between lobule VIIIa and right inferior frontal gyrus (IFG) and left IPL; and between lobule VIIIb and left middle frontal gyrus (MFG), bilateral superior parietal gyrus (SPG), and left precuneus. In addition, within the JME group, the strength of dFC between lobule VIIIb and left precuneus was negatively (*r* = −0.424, *p* = 0.025, Bonferroni correction) related with the frequency score of myoclonic seizures.

**Conclusion:**

In patients with JME, there is a functional dysregulation between the motor cerebellum with DMN and SMN, and the variability of dynamic functional connectivity may be closely associated with the occurrence of motor symptoms in JME.

## 1 Introduction

Juvenile myoclonic epilepsy (JME) accounts for ~9.3% of all epilepsies (1, 2) and is the most common idiopathic generalized epilepsy (IGE) syndrome onset in adolescence and adulthood. Myoclonic seizures are mandatory for diagnosis (3) and may be unilateral or bilateral, frequently involving the upper extremities (4), which occur mostly within the 1st h after awakening and could be facilitated by sleep deprivation (3). The electroencephalograph (EEG) shows 3–5.5-Hz generalized spike-wave and polyspike-wave (5). Generalized tonic–clonic seizures occur in >90% of individuals, and absence seizures occur in one third of cases (6, 7). Multiple types of motor symptom are characteristic of JME, but the pathophysiology of motor symptom seizures in JME remains unknown.

The cerebellum has been found to be associated with the production of several types of myoclonus (8), and its pathological involvement has been demonstrated in benign adult familial myoclonic epilepsy (BAFME) (9), juvenile absence epilepsy (10), non-convulsive partial status epilepsy (11), and JME (12–14). Additionally, it has been hypothesized that the increased inhibitory effect of cerebellum on the basal ganglia-related thalamocortical (BTC) pathway might potentially contribute to the motor symptoms in patients during seizures (15). The functional anatomical topography of the cerebellum indicates that the anterior lobe represents motor function, the vermis is involved in emotion, and the posterior lobe is responsible for complex cognitive functions (16, 17). These functional differences support the existence of a “motor cerebellum,” including lobules V, VI, VIIb, and VIII, projecting to the motor area (precentral gyrus and postcentral gyrus) (18). In temporal lobe epilepsy patients, bilateral cortical thinning was observed in the precentral and postcentral motor regions connected with the anterior and posterior lobe motor areas (lobules VIII) (19); in patients with genetic generalized epilepsy, an asymmetric pattern of reduced cerebellar gray matter volume (GMV) has been found, with greater loss of gray matter in the left “motor” posterior inferior cerebellar regions (VIIIA, VIIIB, and IX) (20). Therefore, in this study, based on previous research (18, 21, 22), we selected lobules I–V, VI, VIIb, VIIIa, and VIIIb as seeds for the motor cerebellum regions of interest (ROI) to explore the relationship between changes in motor cerebellar functional connectivity and the occurrence of motor symptoms in JME.

Furthermore, the functional dysfunction of the frontal lobe, thalamus, and cerebellum with the sensorimotor network (SMN) may be related to the motor symptoms of seizures (23). The activity of high-order association networks such as the default mode network (DMN) plays a crucial role in the generation and spread of generalized epileptic activity (24). Changes in connectivity between DMN regions and the cerebellum may be related to long-term and repetitive functional suspension of the basic brain state during seizures (25). Therefore, in this study, we hypothesize that there is a functional disorder between the motor cerebellum and DMN (26) and SMN (27) in JME patients, which is closely related to the occurrence of motor symptoms in JME patients.

Resting-state functional connectivity (rs-FC) is obtained by visualizing the interactions between blood oxygenation level-dependent (BOLD) signals in different regions of a person's brain during rest (28), while the dFC analysis can be used to capture the variability of the spatiotemporal structure of brain activity and estimate changes in inter-regional synchrony (29). The sliding-window method is widely used in investigating the dFC of various human neurological and psychiatric diseases (30). Therefore, we employed resting-state EEG-fMRI and utilized dFC analysis with a sliding window approach to explore the alterations in dynamic functional connectivity within the motor cerebellum-SMN-DMN and its association with the onset of motor symptoms in JME. Furthermore, we examined the intergroup differences in dFC variability in JME patients and its relationship with clinical variables, such as disease duration, age at disease onset, and frequency score of myoclonic seizures.

## 2 Materials and methods

### 2.1 Participants

The patients with JME were consecutively enrolled from the epilepsy center of the West China Hospital of Sichuan University. The inclusion criteria were as follows: (1) The diagnosis of JME was carried out by two experienced neurologists in accordance with the criteria defined by the International League Against Epilepsy (5) and (2) JME patients had normal development with normal brain MRI, had interictal bilaterally synchronous 4–6 Hz generalized polyspike-wave discharges (GSWDs) with normal background on EEG and had no self-reported cognitive impairment. The exclusion criteria included: (1) MRI results confirming structural pathological changes in brain; (2) Evidence of atypical epilepsy syndrome or secondary epilepsy; and (3) Other neurological, psychiatric, or metabolic illnesses and drug abuse. Ultimately, 36 patients with JME and 30 well-matched HCs were recruited.

The baseline information of participants was recorded including gender, age at disease onset, duration of disease, years of education, frequency score of myoclonic jerks in the last 2 years and the use of antiseizure medications (ASMs). The seizure diaries were used to assess the frequency score of myoclonic jerks in the last 2 years according to the method of previous reports with slight modification (31). In addition, the patients visited our outpatient clinic every 3–6 months and were carefully assessed for the presence of seizures and the use of ASMs. This study was approved by the Institutional Ethics Review Board of the Sichuan University, and all participants and families were given the written informed consent.

### 2.2 EEG and fMRI data acquisition

MRI data were acquired using a 3.0 T magnetic resonance system (Siemens Skyra, Erlangen, Germany). Foam padding and earplugs were used to minimize head movement and scanner noise, respectively. Participants were instructed to keep their eyes closed while remaining awake, and it was confirmed that none had fallen asleep. Functional images were acquired using an echo-planar imaging (EPI) sequence with gradient recalled echo and T2^*^ weighted: 205 volumes; 30 slices; 5 mm thickness; echo time (TE) = 30 ms; repetition time (TR) = 2,000 ms; field of view (FOV) = 24 × 24 cm^2^; matrix size= 64 × 64; in-plane resolution = 2 × 2 mm, and flip angle = 90°. The structural T1-weighted brain images were gained with a three-dimensional-spoiled gradient recalled sequence during the same session: 176 axial slices (thickness: 1 mm, no gap, TE = 2.26 ms; TR = 1,900 ms; FOV = 256 × 256 mm^2^; flip angle = 9°; matrix = 320 × 320; slice thickness = 1 mm). All T1-weighted structural and functional images were visually inspected to ensure that there were no visible artifacts covering the cerebellum.

During the fMRI scans, simultaneous electroencephalography was recorded synchronously using an EEG device (EBNeuro Mizar 40, Italy). The EEG dynamic range was set to ±65.5 mV, and MR artifacts were filtered online using BE-MRI Toolbox software (32). Interictal GSWDs were identified by two experienced epileptologists during the patient scanning process. If interictal GSWDs or seizures occurred during the EEG-fMRI scanning session, the whole session was discarded to avoid the effects of interictal epileptic discharges or ictal events. If either of these two situations occurred, another session will be further performed for the same patient. In total, eight scanning sessions with interictal GSWDs were discarded, and no seizures were reported in all scans.

### 2.3 Preprocessing of MRI data

DPABI V5.0 (http://rfmri.org/dpabi) and SPM8 software package (http://www.fil.ion.ucl.ac.uk/spm) were used to preprocess the functional and structural images. The data volume of patients was consistent. First, the first 10 volumes of fMRI data were discarded, followed by the following steps on the remaining 205 volumes: slice timing correction, realignment of all functional volumes, and then co-registration between the functional volumes and the corresponding T1 volumes. Spatial normalization to Montreal Neurological Institute (MNI) space was performed using Diffeomorphic Anatomical Registration Through Exponentiated Lie algebra (DARTEL) toolbox, with re-sampling to each voxel: 3 × 3 × 3 mm^3^ and used a 4 mm FWMH kernel over the processed time series to smooth spatially and filter temporally (0.01–0.1 Hz). Besides, we used the Friston's 24-parameter mode (33) to minimize head motion confounds. Multiple linear regressions were used to remove confounding covariates, including signals from cerebrospinal fluid and white matter that were regressed out. Additionally, linear trends were regressed to account for drift in BOLD signal. Considering the potential influence of whole-brain signals on functional networks, we did not regress them out in this processing pipeline (34). Participants with maximum displacements > 2 mm or rotations > 2° were excluded. We recorded the head motion of all participants based on the average framewise displacement (FD; Jenkinson value), and participants were excluded from this study if the average FD exceeded 0.2 mm (35). Due to these standards, six patients were excluded. The mean FD of the included subjects did not differ between the JME group and HC group.

### 2.4 Variability analysis of dFC

Based on prior parcellation of the motor cerebellum (lobule I–V, VI, VIIb, VIIIa, and VIIIb) (18, 21, 22), we used predefined bilateral six pairs of 6-mm radius spherical regions as regions of interest (ROIs) for seed-based dFC analysis in all participants. Visual examination was carried out to ensure there was no overlap between each pair of ROIs. A visual inspection was conducted to ensure no overlap between each pair of ROIs. Subsequently, a Hamming sliding window approach was performed based on DPABI, and since the minimum window length should not be <1/f min (defined as the minimum frequency of the time series; 1/0.01 s = 100 s), we chose a window size of 50 TRs (100 s) with a step of 1 TR (2 s) for dFC analysis, which balanced the risk of spurious fluctuations and impediments to dynamic descriptions of temporal variability (36). As a result, 146 sliding-window dFC states were generated. Temporal correlation matrices were created by correlating each truncated time series of the motor cerebellar parcellation with all other voxels within each sliding window. Subsequently, Fisher's *r*-to-*z* transformation was performed on each voxel of the 146 sliding window correlation maps to improve the normality of the correlation distribution. And then, the dFC variability was calculated by calculating the standard deviation of the *z*-value at each voxel of the 146 sliding-window *z*-value maps. Afterwards, further statistical analysis was conducted using dFC maps with *z*-standardization.

### 2.5 Statistical analysis

All analyses were performed using SPSS Software version 21.0. A *P*-value of <0.05 was considered statistically significant. Normality tests and homogeneity of variance were conducted, and ultimately independent samples two-tailed *t*-tests were used to compare demographic and clinical data between the JME group and HC group. A one-sample *t*-test was performed on within-group dFC variability [*p* < 0.001, Gaussian random field (GRF) correction]. To identify significant differences in dFC variability between JME and HC within the sensorimotor network (SMN) and default mode network (DMN) with the motor cerebellum, a linear model was used with age, gender, and mean FD as nuisance covariates. The standard deviation of *z*-values for each voxel served as the dependent variable, with the participant group as the independent variable. The GRF correction was performed with two-tailed voxel *p* < 0.01 and cluster-level *p* < 0.05 (minimum *z* > 2.3; cluster significant: *p* < 0.05, corrected). Using two-sample *t*-test to compare the group differences of head motion through the mean FD. If any seeds of the motor cerebellum showed significant between-group differences in dFC variability, further partial correlation analyses would be conducted to detect potential relationships between dFC variability values and clinical data such as disease duration, age at seizure onset, and frequency scores of myoclonic seizures in JME.

### 2.6 Verification analyses

We conducted validation experiments using two additional window length settings: the first window length was set to 70 TRs (140 s), and the second window length was set to 30 TRs (60 s), and both with a displacement step of 1 TR (2 s).

## 3 Results

### 3.1 Demographic characteristics

**Figure 1:**
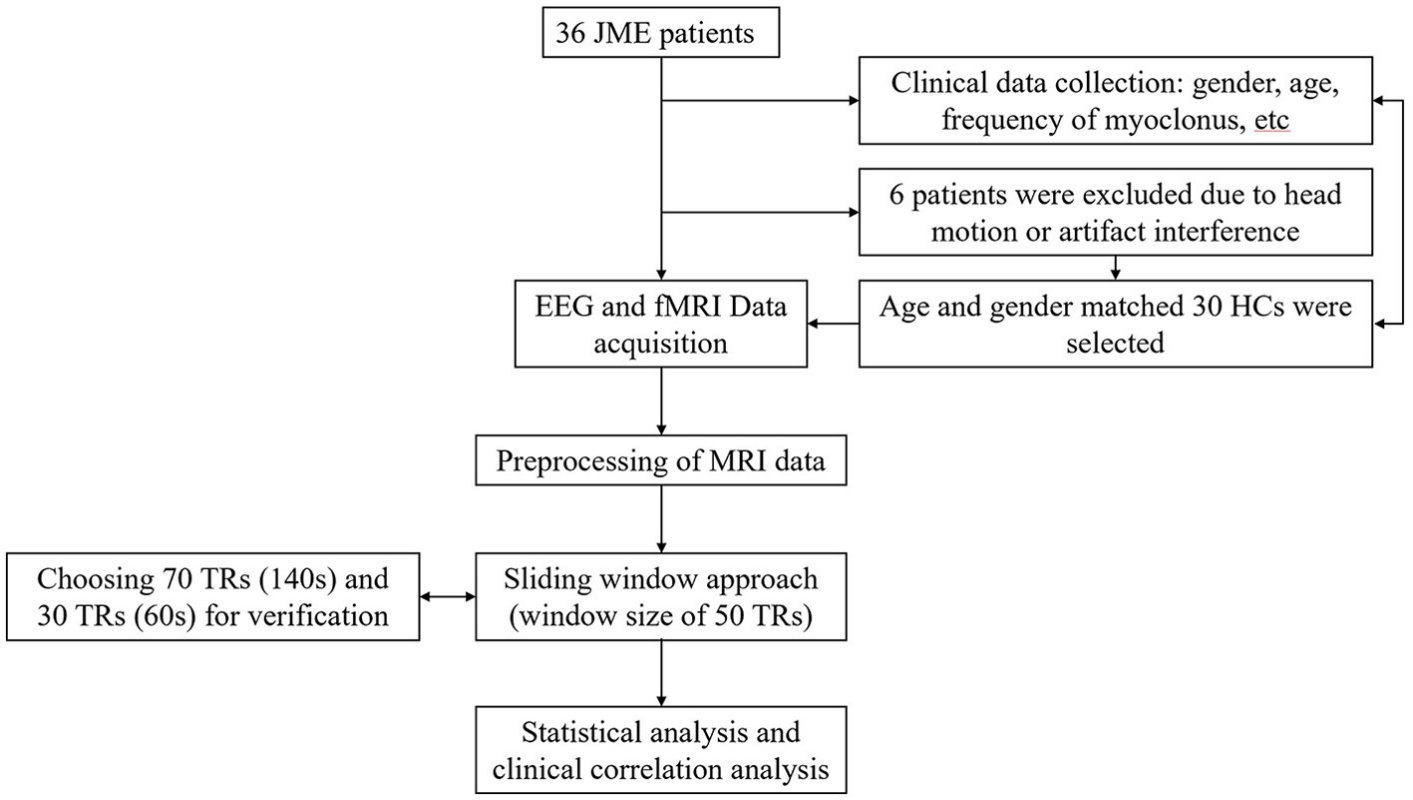
Patient flow. This flowchart illustrates the reasons for patient exclusion and the general research methodology process.

A total of 36 JME patients and 30 HCs were recruited. Due to excessive head motion, six patients were excluded, resulting in a final inclusion of 30 patients and 30 healthy individuals in the study (Figure 1). Among the JME patients, four individuals had not received any medication. Table 1 documented the baseline characteristics of all participants, with no significant differences in age, gender, or mean FD (JME group: 0.105 ± 0.034, HC group: 0.092 ± 0.023, *P*-value = 0.084 > 0.05) between the JME and HC groups.

**Table 1:** Demographic and clinical characteristics of the JME and HC group.

	**JME (*n* = 30)**	**HC (*n* = 30)**	***p*-value**
Gender (male/female)	13/17	13/17	-
Handedness (right/left)	30/0	30/0	**-**
Age of onset ± SD	14.84 ± 3.23	-	-
Age ± SD	19.57 ± 3.98	19.77 ± 3.76	0.842 ^a^
Duration ± SD	4.70 ± 4.51	-	-
EEG	GSWDs	Normal	-
Frequency score of myoclonic seizures	3.33 ± 1.67	-	-
Medication			
Drug naive	4		-
VPA	8		-
LTG	2		-
LEV	14		-
TPM	1		-
VPA+LEV	1		-

^a^Independent sample, two tailed t-test.

JME, Juvenile myoclonic epilepsy; GSWDs, Generalized spike-wave discharges; VPA, Valproate; LTG, Lamotrigine; LEV, Levetiracetam; TPM, Topiramate; SD, standard deviation.

Note: This study used the same subjects as our previous study on the thalamic-cortical circuit in JME patients, so the clinical data characteristics are the same (37).

### 3.2 Dynamic functional connectivity variability results of motor cerebellar seeds

**Figure 2:**
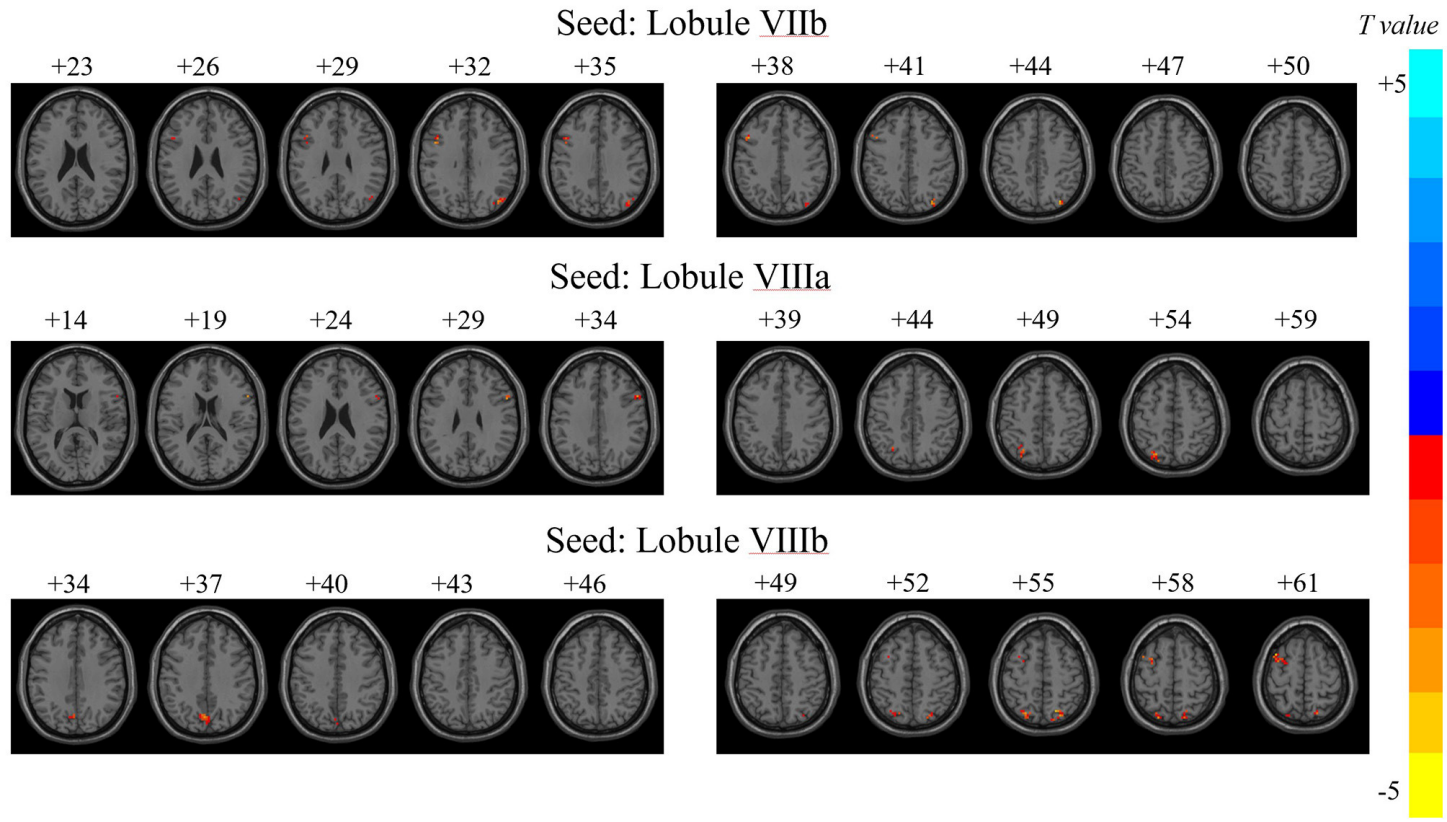
Variability of dFC in motor cerebellar seeds exhibiting between-group differences. *T, t* score of the voxel with peak intensity. The color bar on the right indicated the strength of dFC variability changes, with blue representing decreased dFC variability in JME patients compared to HCs group, and yellow indicating increased dFC variability in JME patients than in HCs.

Significant differences in dFC variability for each motor cerebellar seed between the JME group and HCs were obtained through a general linear model with age, gender, and mean FD as covariates (Table 2 and Figure 2, *z*_min_ > 2.3; cluster significance: *p* < 0.05, GRF corrected). As a result, in comparison to HCs, the JME group exhibited increased dFC between the motor cerebellum with SMN and DMN. Specifically, connectivity between lobule VIIb and left precentral gyrus and right inferior parietal lobule (IPL) increased; dFC between lobule VIIIa and these two structures right inferior frontal gyrus (IFG) and left IPL increased; and connectivity between lobule VIIIb and four structures including left middle frontal gyrus (MFG), bilateral superior parietal gyrus (SPG), and left precuneus increased.

**Table 2:** Dynamic FC variability differences between JME patients and HC (minimum *z* > 2.3; voxel level: *p* < 0.01, cluster significance: *p* < 0.05, GRF corrected).

**Seeds**	**Significant regions**	**BA**	**Voxels**	**MNI**			***T***	**Comparisons**	***P***
				***x***	***y***	***Z***			
VIIb	L PCG	44	20	−45	15	39	4.103	Patients>HCs	0.00014
	R IPL	39	29	33	−75	45	4.636	Patients> HCs	0.00002
VIIIa	R IFG	44	18	54	12	27	3.905	Patients>HCs	0.00025
	L IPL	7	29	−24	−69	54	3.684	Patients>HCs	0.00053
VIIIb	L Precuneus	7	21	−3	−72	36	3.775	Patients>HCs	0.00039
	L MFG	6	25	−39	12	60	4.320	Patients> HCs	0.00006
	L SPG	7	21	-−24	−69	54	3.881	Patients>HCs	0.00028
	R SPG	7	31	18	−66	54	4.399	Patients>HCs	0.00005

T, t score of the voxel with peak intensity; JME, Juvenile myoclonic epilepsy; dFC, dynamic functional connectivity; BA, Brodmann Area; MNI, Montreal Neurological coordinate; PCG, precentral gyrus; IPL, inferior parietal lobule; MFG, middle frontal gyrus; IFG, inferior frontal gyrus; SPG, superior parietal gyrus; L (R), left (right) hemisphere.

The numbers at the top of the image represented the MNI *z*-coordinates.

### 3.3 The relationship between dFC of the motor cerebellum and clinical variables in JME group

**Figure 3:**
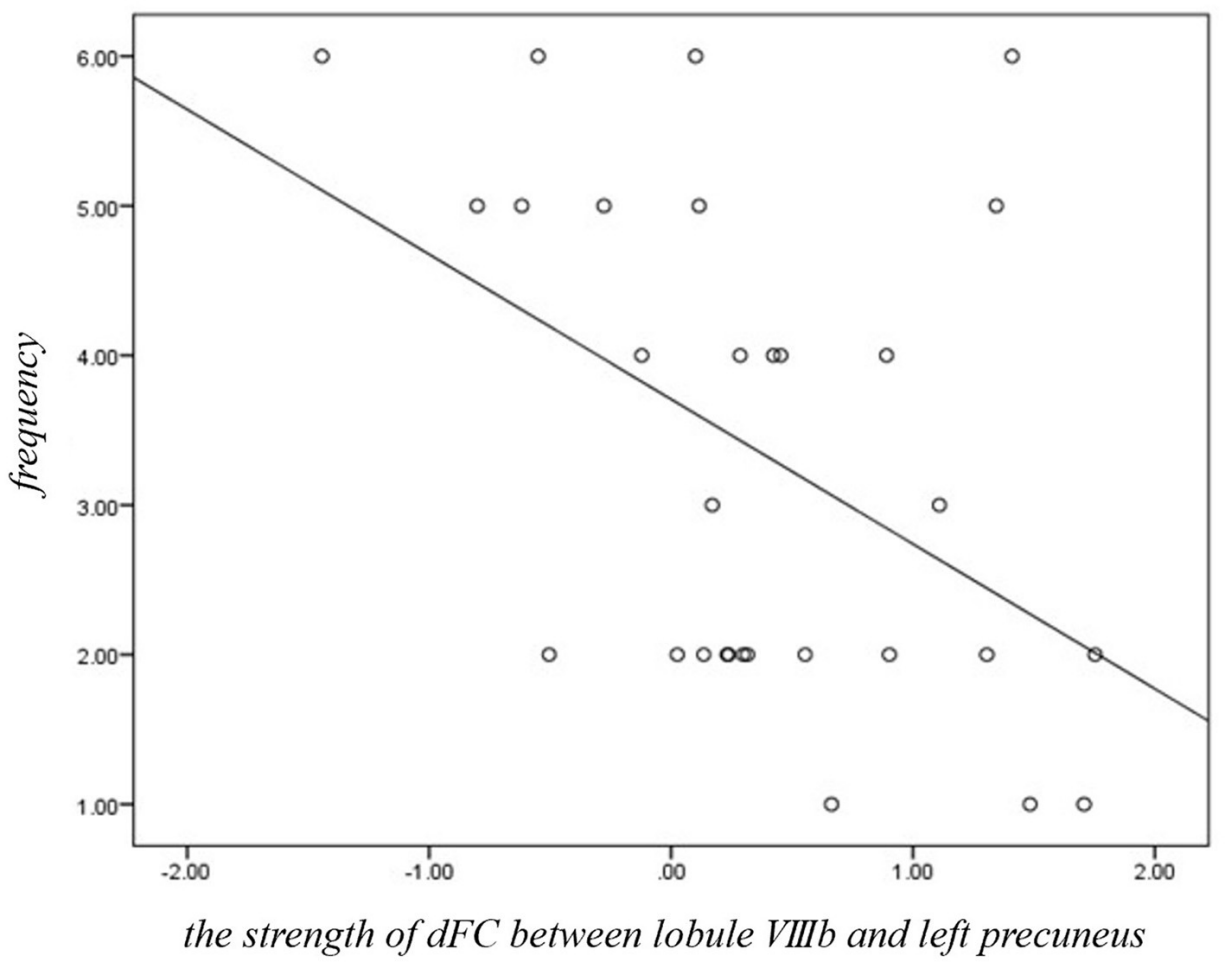
Significant correlations between dFC of motor cerebellum and clinical variables in JME group. The strength of dFC between lobule VIIIb and left precuneus was negatively (*r* = −0.424, *p* = 0.025, Bonferroni correction) related with the frequency score of myoclonic seizures in JME.

Within the JME group, the strength of dFC between lobule VIIIb and left precuneus was negatively (Figure 3, *r* = −0.424, *p* = 0.025, Bonferroni correction) related with the frequency score of myoclonic seizures. No significant relationships were identified between the other significantly different dFC variability of motor cerebellar seeds and any clinical characteristics including the age at disease onset, disease duration, or years of education in JME patients.

### 3.4 Verification results

We compared the dFC variability of JME patients with 70 TRs and 30 TRs sliding window lengths, as well as the group differences in dFC between JME and HCs.

In validation experiments, results from two different window lengths slightly deviated from our main findings, yet the variability of dFC predominantly manifested between the motor cerebellum and SMN and DMN (detailed in Supplementary Figures 1, 2, Supplementary Tables 1, 2).

## 4 Discussion

The present study firstly investigated the dFC of the motor cerebellum in patients with JME. The major findings were as follows. First, patients with JME showed increased dFC between the motor cerebellum with SMN and DMN. Besides, the strength of dFC between lobule VIIIb and left precuneus was negatively related with the frequency score of myoclonic seizures. These results suggest that the motor cerebellum has characteristic dFC alterations in JME, and these dFC alterations may provide the first evidence for the dynamic and wide involvement of the motor cerebellum in the seizure of motor symptoms in JME.

The cerebellum is not only a gateway for neural regulation by the cerebrum (38), but also stimulation signals originating from the cerebellum could alter the functional connections of distant cerebral cortices (39, 40). Different cerebellar regions have distinct functions, with the anterior cerebellum involved in feedforward motor control, while the posterior half could transform visual feedback for precise motor adjustments (41). Based on functional distinctions or preferential connectivity, the cerebellum could be further divided into a “motor” region, which includes lobules V, VI, VIIb, and VIII, and a “cognitive” region that comprises Crus I and II within lobule VIIa (18, 42, 43). Therefore, it could be demonstrated that using dFC analysis of the five seeds of the motor cerebellum (lobules I–V, VI, VIIb, VIIa, and VIIIb) can better understand the role of the motor cerebellum in the onset of motor symptoms in JME.

Motor symptom seizures such as myoclonic seizures are characteristic seizure types of JME. Researchers have demonstrated that the cerebellum receives extensive information widely from the cerebral cortex (44), and projects it to the primary motor cortex through the thalamus to regulate movement (45). A proportion of epileptic seizures may be directly triggered by the cerebellum (46), and the most common symptom of seizures caused by lesions located in the cerebellum is myoclonic seizures (8). Additionally, increased connectivity between the cerebellum and the precentral gyrus (47, 48) (regulating movement) as well as the insula (49) and amygdala (50) (regulating emotions) might bypass the executive control network (ECN), which could help explain the involuntary movements and motor manifestations during epileptic seizures (44, 51). The inferior parietal lobule (IPL) plays an important role in the continuous movement of skilled actions and the associated control processes (52), whereas during parietal lobe seizures, there is an increase in FC between the premotor cortex and the superior parietal lobule (SPG) (53). In our study, JME patients showed an increased connectivity between the motor cerebellum and the cortex involved in SMN such as IPL and precentral gyrus. SMN, as a sensor of the brain, is responsible for perceiving physical inputs and initiating physical reactions (54). Therefore, these results indicate that the alterations of the dFC between the motor cerebellar with SMN may be associated with motor symptoms in JME patients.

Prolonged recurrent discharges may impair the overall function of DMN (24), and dysfunction of DMN may indicate a disturbance of fundamental brain functions (55), which has been validated in neurological disorders such as schizophrenia (56) and Parkinson's disease (57). Studies have found that functional connectivity within the DMN is abnormal in IGE patients, irrespective of the presence or absence of GSWDs (58). The cerebellar output to the cerebral cortex is predominantly inhibitory (59), and an increased excitatory cerebellar outflow to the cerebral cortex may attenuate seizure activity (60). Previous studies have found that in IGE patients, the thalamus and cerebellum can weaken the interaction between DMN and SMN (61). The reduction in cerebellar inhibitory function has been identified as a cause of somatosensory enhancement, which could be a reason for motor abnormalities in epilepsy (62). In our study, we found that the increased functional connectivity strength between the motor cerebellum (lobule VIIIb) and DMN (precuneus) was negatively correlated with the fraction of myoclonic seizure frequency in the JME patient group. According to this, we propose a reasonable speculation that the motor cerebellum impacts various targets by multiply pathway which may contribute to the motor symptoms in JME, or which are inferred to imply a subdued unrestrictive effect of the motor cerebellum on the cerebral cortex in JME and could be the secondary compensation mechanism. In summary, our results further support and supplement the changes in the connectivity of the motor cerebellum with DMN and SMN, and are involved in the abnormal processing of sensory-motor information in JME.

Cerebellum has been considered involved in high-order cognitive network. Previous studies showed that the cerebellum exhibits intrinsic connectivity with the DMN in healthy participants (63). The precuneus is the core node of DMN (64), and its prominent functions are emotional processing (65) and interpersonal communication (66). Increased functional connectivity in the cerebellar-thalamo-cortical (CTC) pathway with the precuneus has been found in IGE patients (15), consistent with our study, suggesting aberrant interactions between the motor cerebellum and the default mode network (DMN), which may contribute to cognitive dysfunction in JME patients, yet further exploration is still required.

## 5 Limitations

Our study has limitations which are mentioned below. First, the sample size is relatively modest in our group; the findings of this work should be replicated and validated with a larger sample. Secondly, the effect of medication response on dFC variability was not explored in our study, and this work should include validation with more JME patients who have not used antiseizure medications (ASMs). Thirdly, the direct relationship between dFC and cognitive dysfunction could not be assessed; further, we will quantify cognitive dysfunction in patients using cognitive function scales and specialized cognitive assessment methods. Educational information might influence cognitive function, yet the education status of the subjects was not documented in this study, thus precluding the avoidance of potential impacts of this factor on the results. In future investigations delving into the relationship between motor cerebellum and cognitive function in JME patients, we will incorporate the lessons learned from this experience. Additionally, it is imperative that we acknowledge the relatively permissive statistical threshold employed in our analyses within this study, which may be conducive to an elevated rate of Type I errors. Finally, although procedures were carried out to minimize the impact of head motion on dFC results, the effects cannot be completely eliminated. In future research, we will expand the sample size and use big data models as well as data-driven approaches to further explore the pathophysiological mechanisms of JME, drug responses, and more.

## 6 Conclusion

In patients with JME, there is a functional dysregulation between the motor cerebellum with DMN and SMN, and the variability of dynamic functional connectivity may be closely associated with the occurrence of motor symptoms in JME.

## Data availability statement

The original contributions presented in the study are included in the article/Supplementary material, further inquiries can be directed to the corresponding authors.

## Ethics statement

The studies involving humans were approved by the Institutional Ethics Review Board of the Sichuan University. The studies were conducted in accordance with the local legislation and institutional requirements. Written informed consent to participate in this study was provided by the patients/participants or patients/participants' legal guardian/next of kin. Written informed consent was obtained from the individual(s), and minor(s)' legal guardian/next of kin, for the publication of any potentially identifiable images or data included in this article.

## Author contributions

MY: Writing – review & editing, Writing – original draft, Methodology, Investigation, Data curation. YZ: Writing – review & editing, Methodology, Data curation. TZ: Writing – review & editing, Methodology, Formal analysis, Data curation. HZ: Writing – review & editing, Methodology, Investigation, Data curation. JR: Writing – review & editing, Methodology. DZ: Writing – review & editing, Visualization, Supervision, Conceptualization. TY: Writing – review & editing, Supervision, Software, Investigation, Conceptualization.
