# Supplementary material for: Altered dynamic functional connectivity of motor cerebellum with sensorimotor network and default mode network in juvenile myoclonic epilepsy
Source: Front Neurol. 2024 Jun 6;15:1373125. doi: 10.3389/fneur.2024.1373125 (PMC11187336; doi:10.3389/fneur.2024.1373125)
Supplement: Supplementary file 1 [file Data_Sheet_1.docx]

1. **Validation results for the 70 TRs sliding window length**


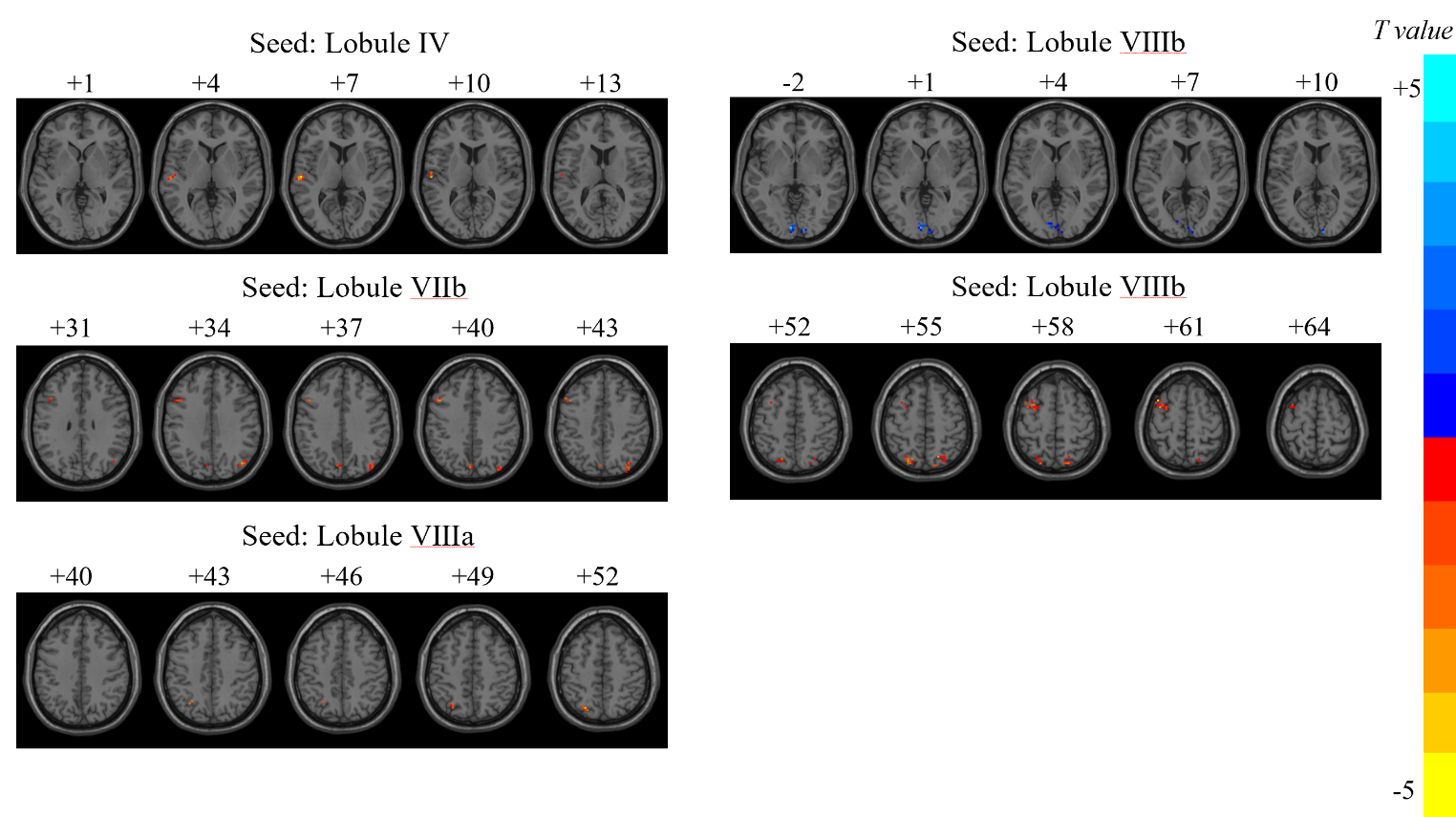


**Figure s-1 Variability of dFC in motor cerebellar seeds exhibiting between-group differences (70TRs)**

Note: The color bar on the right indicated the strength of dFC variability changes, with blue representing decreased dFC variability in JME patients compared to HCs group, and yellow indicating increased dFC variability in JME patients than in HCs. The numbers at the top of the image represented the MNI z-coordinates.

| Seeds | Significant regions | BA | voxels | MNI | | | *T* | Comparisons | *P* |  |
| --- | --- | --- | --- | --- | --- | --- | --- | --- | --- | --- |
|  |  |  |  | x | y | z |  |  |  |  |
| IV  Ⅶb  Ⅷa  Ⅷb | L STG  L PCG  R IPL  L Precuneus  L IPL  L MFG  L SPG  R SPG  L Calcarine  R Calcarine | 22  6  39  7  7  8  7  7  17  18 | 18  19  49  19  18  34  19  19  26  5 | -57  -45  42  -3  -24  -39  -24  18  -6  13 | -18  15  -72  -78  -69  12  -72  -66  -90  -94 | 6  39  33  39  51  60  54  54  -3  -2 | 4.817  4.359  4.147  3.972  3.864  4.866  3.884  4.039  -4.375  -3.978 | Patients> HCs  Patients>HCs  Patients>HCs  Patients>HCs  Patients> HCs  Patients>HCs  Patients>HCs  Patients>HCs  Patients<HCs  Patients<HCs | 0.00001  0.00006  0.00012  0.00021  0.00030  0.00001  0.00028  0.00017  0.00005  0.00019 |  |

**Table s-1**. **Dynamic FC variability differences between JME patients and HC (70TRs) (minimum z > 2.3; voxel level: p<0.01, cluster significance: p < 0.05, GRF corrected).**

Abbreviation: JME, Juvenile myoclonic epilepsy; FC, functional connectivity; BA, Brodmann Area; MNI, Montreal Neurological coordinate; STG：superior temporal gyrus; PCG: precentral gyrus; IPL, inferior parietal lobule; MFG: middle frontal gyrus; IFG: inferior frontal gyrus; SPG: superior parietal gyrus; L (R), left (right) hemisphere.

1. **Validation results for the 30 TRs sliding window length**


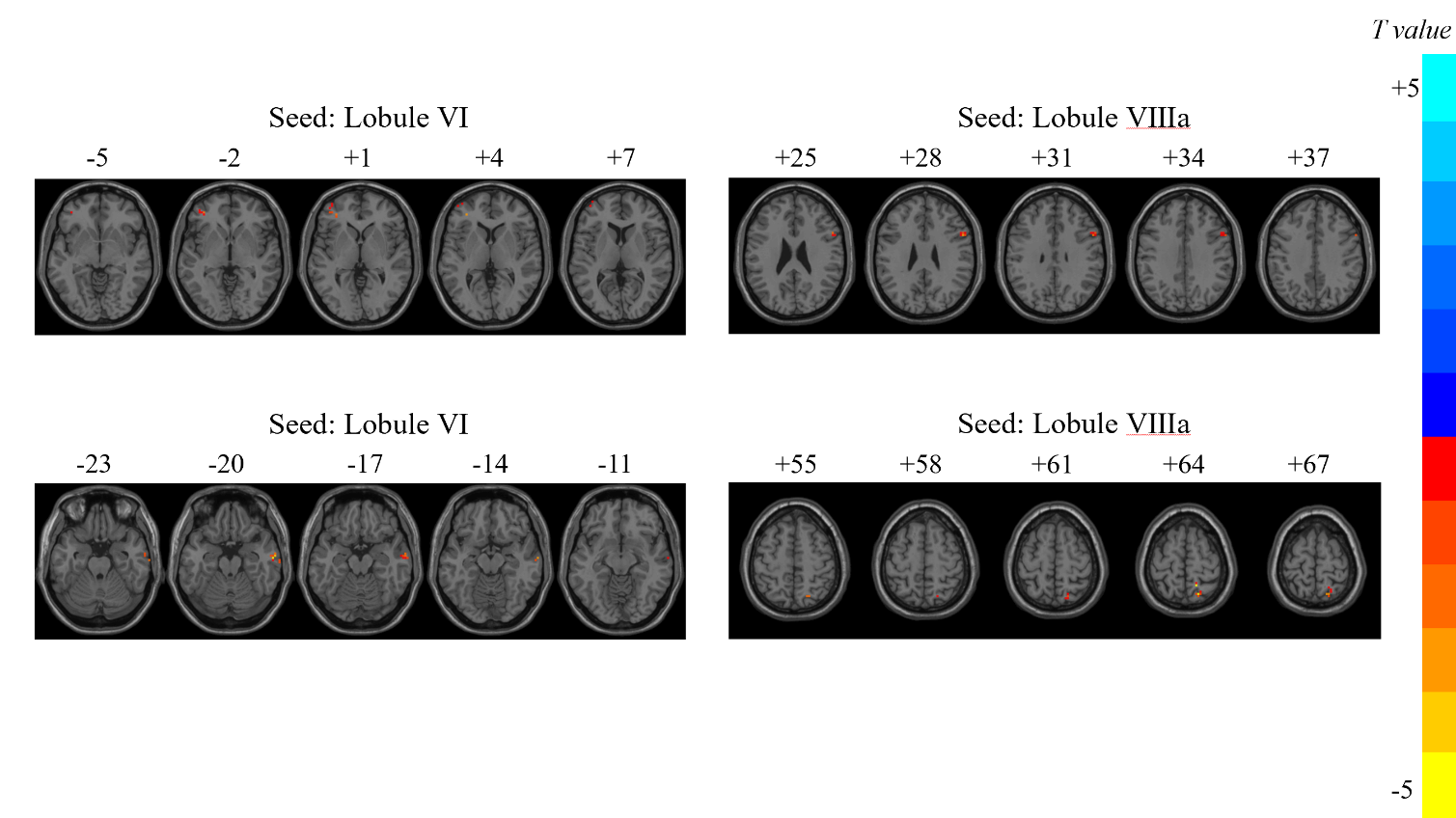


**Figure s-2 Variability of dFC in motor cerebellar seeds exhibiting between-group differences (30TRs)**

Note: The color bar on the right indicated the strength of dFC variability changes, with blue representing decreased dFC variability in JME patients compared to HCs group, and yellow indicating increased dFC variability in JME patients than in HCs. The numbers at the top of the image represented the MNI z-coordinates.

| Seeds | Significant regions | BA | voxels | MNI | | | *T* | Comparisons | *P* |  |
| --- | --- | --- | --- | --- | --- | --- | --- | --- | --- | --- |
|  |  |  |  | x | y | z |  |  |  |  |
| VI  Ⅷa  Ⅷb | L MFG  R MTG  R IFG  R SPG | 47  21  44  7 | 18  22  27  21 | -33  60  54  18 | 42  -12  12  -63 | 3  -21  27  63 | 3.936  4.417  3.925  5.036 | Patients>HCs  Patients>HCs  Patients>HCs  Patients> HCs | 0.00024  0.00005  0.00024  0.00001 |  |

**Table s-2**. **Dynamic FC variability differences between JME patients and HC (30TRs) (minimum z > 2.3; voxel level: p<0.01, cluster significance: p < 0.05, GRF corrected).**

Abbreviation: JME, Juvenile myoclonic epilepsy; FC, functional connectivity; BA, Brodmann Area; MNI, Montreal Neurological coordinate; MFG: middle frontal gyrus; MTG: middle temporal gyrus; IFG: inferior frontal gyrus; SPG: superior parietal gyrus; L (R), left (right) hemisphere.
